# Seroprevalence of SARS-CoV-2 and Risk Assessment Among Healthcare Workers at a Dedicated Tertiary Care COVID-19 Hospital in Delhi, India: A Cohort Study

**DOI:** 10.7759/cureus.20805

**Published:** 2021-12-29

**Authors:** Pragya Sharma, Rohit Chawla, Saurav Basu, Sonal Saxena, Warisha Mariam, Pradeep Kumar Bharti, Shivani Rao, Neha Tanwar, Anisur Rahman, Mohammad Ahmad

**Affiliations:** 1 Community Medicine, Maulana Azad Medical College, New Delhi, IND; 2 Microbiology, Maulana Azad Medical College, New Delhi, IND; 3 Academics, Indian Institute of Public Health, New Delhi, IND; 4 Health and Research Emergency, World Health Organization, New Delhi, IND; 5 Research, World Health Organization, New Delhi, IND

**Keywords:** india, seroconversion, seroprevalence, covid-19, sars-cov-2

## Abstract

Background

Healthcare workers (HCWs) have a substantially higher risk of Covid-19 infection but there is a paucity of information on the risk factors of disease transmission in high-burden real-world settings.

The study objective was to determine the seroprevalence of SARS-CoV-2 among healthcare workers in a high-burden Covid-19 setting and to estimate the incidence and identify the risk factors of infection.

Methods

This was a prospective observational cohort study amongst doctors and nurses working at a dedicated Covid-19 tertiary care government hospital in Delhi, India. A baseline blood sample (2-3ml) was collected from all the participants to test for the presence of total SARS-CoV-2 antibodies. The HCWs that were seronegative (non-reactive) at baseline were followed-up for ≥21≤28 days with the collection of a second blood sample to assess for the incidence of SARS-CoV-2 infection.

Results

A total of 321 (51.3%, 95% C.I 47.4, 55.3) HCWs were detected with SARS-CoV-2 antibodies on baseline examination. The seroprevalence, when adjusted for assay characteristics, was 54.5% (95% C.I 50.3, 58.6). On bivariate analysis, SARS-CoV-2 antibody positivity lacked statistically significant association with either age, sex, occupation, cumulative duty duration, and smoking status. The incidence of seroconversion in the baseline seronegative cohort on follow-up after 21-28 days was observed in 35 (14.9%) HCWs (n=245). Furthermore, the self-reported adherence to infection prevention and control measures did not show a statistically significant association with antibody positivity in the HCWs, neither at baseline nor on follow-up.

Conclusions

The high risk of SARS-CoV-2 transmission in HCWs may be substantially reduced by adherence to Infection Prevention Control (IPC) and protective measures.

## Introduction

The coronavirus disease 2019 (COVID-19) pandemic caused by the severe acute respiratory syndrome coronavirus 2 (SARS-CoV-2) virus has caused more than 189,257,791 cases and 4,070,341 deaths worldwide. In India, the SARS-CoV-2 has been attributed to causing in excess of 31,026,829 cases and 412,531 deaths to date [1, 2].

Healthcare workers (HCWs) including doctors, nurses and other paramedical staff constituting the frontline workforce of a medical health system inevitably experience prolonged periods of direct contact with COVID-19 patients during patient care and management, especially during procedures involving aerosol generation [3, 4]. The prolonged exposure to the virus among HCWs may increase their susceptibility to contracting the SARS-CoV-2 infection compared to the general population. However, the risk of infection may be reduced by adherence to the recommended non-pharmaceutical interventions including social distancing, masking, and hand hygiene, in addition to the appropriate use of personal protective equipment [5].

Safeguarding HCWs against SARS-CoV-2 represents a pivotal public health goal to ensure the capacity of the healthcare system to deliver uninterrupted services to the affected population, and to prevent transmission of infection from infected HCWs to their colleagues, patients, and the community [6, 7].

The detection of SARS-CoV-2 antibodies in individuals signifies the presence of a previous infection from the virus with the ensuing clinical spectrum being mostly asymptomatic or mild presentation in 85% cases, with moderate and severe presentation in the remainder [8]. SARS-CoV-2 seroprevalence surveys screen for antibodies in individuals to assess for the spread of infection in the population and for identifying sociodemographic and clinical factors associated with the risk of infection [9]. Galanis et al on pooled analysis from worldwide studies observed the seroprevalence of antibodies in HCWs as 8.7% (95% CI 6.7-10.9%), with risk factors of infection recognized to be suboptimal hand hygiene before and after patient contact, longer work hours, and improper personal protective equipment (PPE) usage [10]. Seroprevalence studies in HCWs have indicated the presence of SARS-CoV-2 antibodies ranging from 4% to 22% globally, although there are very few studies that have been conducted in India and none with a prospective design to estimate the incident risk [11-16].

The World Health Organization (WHO) has recommended the conduct of serial serosurveys to assess the extent of COVID-19 infection in a specified population and understand the risk factors and the dynamics of infection transmission [9]. Estimating the seroprevalence, trends, and identifying the risk factors of infection in a high burden Covid-19 hospital setting is necessary for understanding the factors contributing to virus transmissibility, especially in the local geographic context considering the prevalent virus strains.

With this background, we conducted this study with the objectives of determining the seroprevalence of SARS-CoV-2 among healthcare workers in a dedicated tertiary care government hospital, and ascertaining the incidence and risk factors of infection.

## Materials and methods

Study design, setting, and participants

This was a prospective observational cohort study of healthcare workers (HCWs) working in a dedicated 2200-bedded COVID-19 Care Centre in a metro city, which has managed over 20,000 moderate-to-severe COVID-19 cases since April 2020 [17]. The data were collected from 2 December 2020 to 25 May 2021.

The study participants were HCWs comprising either doctors or nurses who were involved in the provision of care to any laboratory-confirmed COVID-19 patient, either through real-time polymerase chain reaction (PCR) or antigen assay, following their admission and navigation through various departments of the hospital as part of the continuum of care. We included those HCWs who were currently deployed at any COVID-19 ward or intensive care unit of Lok Nayak Hospital (LNH) and were involved in the clinical management of any laboratory-confirmed COVID-19 patient. The participants were enrolled irrespective of the presence of any symptoms of suspected COVID-19-like illness at the time of recruitment. However, the HCWs who reported any laboratory-confirmed COVID-19 case among their close contacts at the time of recruitment were excluded. Doctors and nurses were posted for shifts of 15-day duties at the ICUs and wards followed by a minimum duration of 7 days of quarantine usually offered at designated hotels and hostels.

Outcome, sample size, and sampling strategy

The primary outcome was the proportion of study participants who have serological evidence (anti-SARS-CoV-2 total antibody) of SARS-CoV-2 infection at baseline, and the incidence of SARS-CoV-2 infection in the seronegative cohort during follow-up.

The sample size for the cross-sectional analysis is adequate considering the seroprevalence of IgG SARS-CoV-2 antibody in doctors and nurses as 7.7% based on a previous study in the same institution during August-September 2020, at 95% confidence levels, 2% absolute precision, and 5% non-response was estimated to be 630 [18]. For the cohort analysis, at 95% confidence level, 80% power, assuming the ratio of unexposed and exposed to be 1 (since exposure could not be determined in those using PPE), expecting infection in the exposed group (high-risk HCWs) to be 5.4% and unexposed group (low-risk HCWs) to be 1.2% from a study in Germany, and considering 10% loss to follow-up, the sample size was calculated to be 566 on applying the Fleiss formula [19].

The sampling strategy was based on simple random sampling at the ward/ICU level for the selection of the study participants. There are 32 wards and intensive care units in LNH with ~1400 doctors and nurses involved in the management of COVID-19 patients who are posted for duty on a rotation basis as per the duty roster. HCWs, including doctors and nurses meeting the eligibility criteria, were recruited from these wards and ICUs. On each day of data collection, one ward/ICU was selected by simple random sampling using computer-generated random numbers. The sampling frame was generated from the doctor and nurse’s duty roster for the fortnight after excluding previously enrolled or non-responsive HCWs. Within the ward, on each day, a total of eight eligible HCWs were recruited in the study through the lottery method with additional HCWs contacted in case of non-response. 

Data collection

Data instruments used in this study included an interview schedule to collect sociodemographic information of the participants. The information on adherence to infection prevention and control measures and contact with and type of exposure to the COVID-19 patients following their admission to the health care facility were measured at baseline and on follow-up using Forms 1 and 2, respectively. The symptom diary captured the onset and duration of symptoms related to COVID-19-like illness among the seronegative participants at baseline during a period of telephonic follow-up lasting at least 21 days until the follow-up sample collection. A participant was considered as symptomatic if they had any one of the following symptoms for a period of ≥1 day: fever, sore throat, cough, runny nose, shortness of breath, chills, vomiting, nausea, diarrhoea, conjunctivitis, rash, headache, muscle aches, joint ache, loss of appetite, loss of smell, nose bleed, and fatigue.

Laboratory evaluation and processing

We collected a baseline blood sample (2-3ml) from all the study participants to test for the presence of total SARS-CoV-2 antibodies. The HCWs that were seronegative (non-reactive) were followed-up for ≥21≤28 days with the collection of a second blood sample to assess the incidence of SARS-CoV-2 infection. All the staff involved in the collection, transportation, processing, and storage of specimens were trained in safe handling practices and spill decontamination procedures. For each blood sample collected, the time of collection, the conditions for transportation, and the time of arrival at the laboratory were recorded. Specimens were sent to the laboratory immediately upon collection.

Anti-SARS-CoV-2 total antibody was detected in the serum samples using Wantai^TM^ SARS-CoV-2-Ab ELISA kit (Beijing Wantai Biological, Beijing, China), an enzyme-linked immunosorbent assay (ELISA) for the qualitative detection of total antibodies to SARS-CoV-2 virus in human serum or plasma specimens. The assay is a two-step incubation antigen “sandwich” enzyme immunoassay, which uses polystyrene microwell strips pre-coated with recombinant SARS-CoV-2 antigen. The specificity of the assay kit was 100% and the sensitivity was 94.7% as per kit literature.

Statistical analysis

Data was managed in EpiData manager using EpiData entry client (single-entered) (EpiData Association, Odense, Denmark). The data was analysed using STATA 14 (StataCorp, College Station, USA). The results were expressed in frequency and proportions for categorical variables, mean and standard deviation for normally distributed, and median and interquartile range for non-normal data representation of the continuous variables. Seroprevalence estimates were reported as proportions with 95% confidence intervals. The apparent (crude) seroprevalence was adjusted for the assay characteristics using the formula for True (Adjusted) prevalence = Apparent seroprevalence + (Specificity - 1) / [Specificity + Sensitivity - 1] [20]. The association between categorical variables was assessed using the Chi-square test. A p-value < 0.05 was considered statistically significant.

Ethical considerations

The study was approved by the Institutional Ethics Committee (F.1/IEC/MAMC/(79/07/2020/No203). Written and informed consent was obtained from all the study participants. The reports of antibody testing were intimated to each participant individually with confidentiality maintained at all stages of the study.

## Results

Participant characteristics

**Figure 1:**
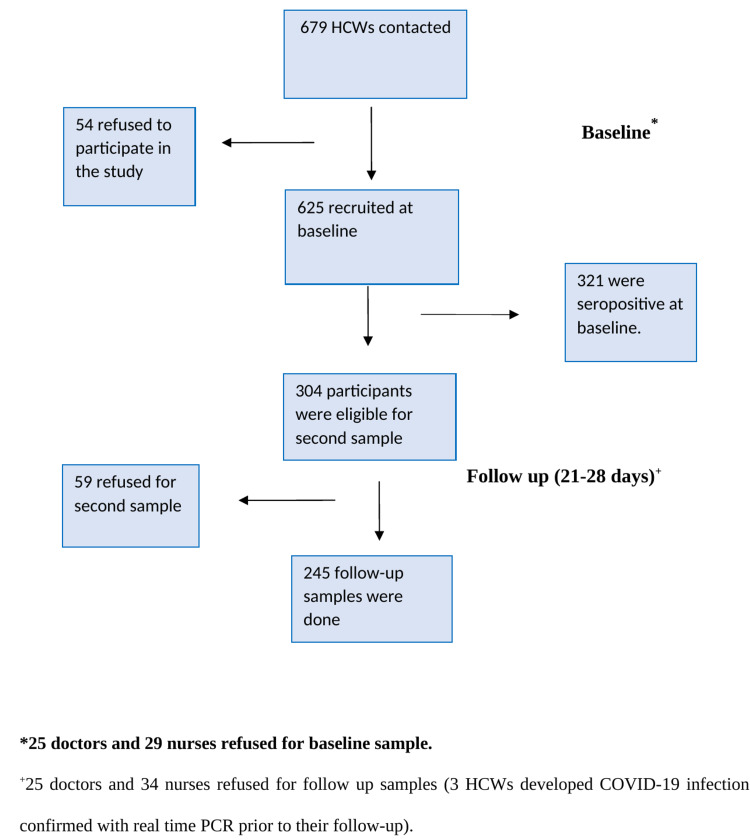
Flow diagram of the recruitment of participants

We recruited a total of 625 HCWs participants comprising 202 doctors and 423 nurses with a net response rate of 92.7% (Figure 1).

The mean (SD) age of the HCWs was 36.7 (9.5 years) including 253 (40.5%) males and 372 (59.5%) females. Faculty doctors comprised only 18 (2.9%) while senior and mid-level nurses comprised 132 (21.1%) and 213 (34.1%) of the participants, respectively. The cumulative duration of prior ward or ICU-related COVID-19 duty in the previous 6 months was <15 days in 392(62.9%), between 15-60 days in 80 (12.5%), and ≥61 days in 153 (24.5%) HCWs. A total of 136 (21.8%) participants had a history of COVID-19 infection diagnosed with real-time PCR or antigen tests, of which 26 (19.1%), 82 (60.3%), 22 (16.2%), and 6 (4.4%) HCWs had asymptomatic, mild, moderate and severe disease, respectively. The most common pre-existing chronic disease conditions in the HCWs were obesity 42 (6.7%), lung disease 40 (6.4%), diabetes 27 (4.3%), heart disease 22 (3.5%), and liver disease 5 (0.8%).

Seroprevalence of SARS-CoV-2 and its predictors

A total of 321 (51.3%, 95% C.I 47.4, 55.3) HCWs were detected with total antibodies to SARS-CoV-2 on baseline examination. The seroprevalence, when adjusted for assay characteristics, was 54.5% (95% C.I 50.3, 58.6).

On bivariate analysis, SARS-CoV-2 antibody positivity lacked statistically significant association with either age, sex, occupation, cumulative duty duration, or smoking status. However, the HCWs with a history of prior COVID-19 had 11.66 times higher odds of seropositivity. Participants who had received vaccination with two doses of either COVID-19 vaccine (n=43) had 100% seroprevalence at baseline, while those who received a single vaccine dose also had 5.8 times higher odds of seropositivity compared to the non-vaccinated HCWs. The seropositivity amongst the HCWs working in the gynaecology and intensive care departments was also higher compared to the other departments. (Table 1)

**Table 1:** Factors associated with seropositivity (SARS-CoV-2 total antibody positive) in study participants at baseline (N=625) ILI: influenza-like illness; ENT: ear nose throat

Characteristic	Total	SARS-CoV-2 Seropositive	Unadjusted odds	p-value
	No.(%)	No. (%)		
Age				
Mean (SD)	36.8 (10.1)		0.99 (0.97, 1.00)	0.104
Sex				
Male	253 (40.5)	131 (51.8)	1	0.863
Female	372 (59.5)	190 (51.1%)	0.97 (0.71, 1.34)	
Occupation				
Doctor	203 (32.48)	111 (54.7)	1	0.25
Nurse	422 (67.5)	210 (49.8)	0.82 (0.59, 1.15)	
Duty duration				
<2 months	472 (75.5)	251 (53.2)	1	0.081
≥2 months	149 (23.8)	67 (45.0)	0.72 (0.50, 1.04)	
History of Covid-19 infection				
Present	136 (21.8)	121 (89.0)	11.66 (6.62, 20.53)	<0.001
Absent	489 (78.2)	200 (40.9)	1	
ILI Symptoms				
Present	64 (10.2)	34 (53.1)	1	0.766
Absent	561 (89.8)	287 (53.1)	1.08 (0.64, 1.82)	
Smoker				
Yes	22 (3.5)	14 (63.6)	1.69 (0.70, 4.08)	0.246
No	603 (96.5)	307 (50.9)	1	
Vaccination status				
Complete	43 (6.9)	43 (100)	110 (6.7, 1799.6)	
Partial	56 (8.9)	46 (82.1)	5.8 (2.9, 11.9)	<0.001
Non-vaccinated	526 (84.2)	232(44.1)	1	
Department				
Casualty	43	23 (53.5)	-	-
ENT	6	3 (50)	-	-
Emergency	29	9 (31)	-	-
Gynaecology	69	45 (65.2)	-	-
ICU/Anaesthesia	134	74 (55.2)	-	-
Medical Ward	72	31 (43)	-	-
Neurology	16	9 (56.2)	-	-
Surgery	138	64 (46.4)	-	-
Orthopaedics	14	8 (57.1)	-	-
Others	48	26 (54.2)	-	-

**Figure 2:**
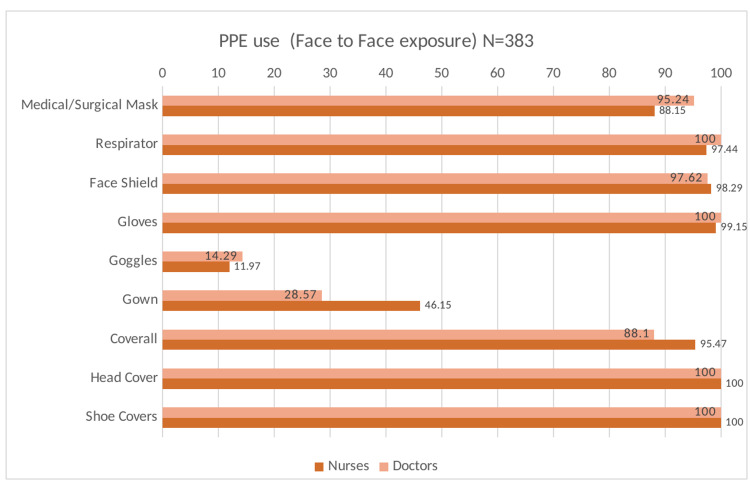
Distribution of utilization of PPE during various exposure to COVID-19 infected patients PPE: Personal protective equipment

**Figure 3:**
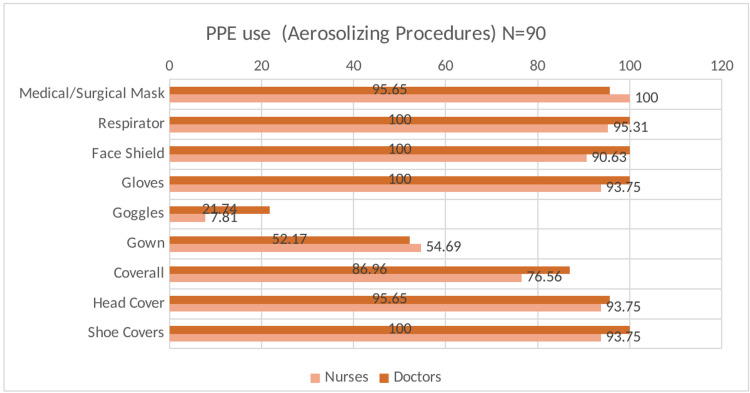
Distribution of utilization of PPE while conducting aerosolized procedure to COVID-19 infected patients PPE: Personal protective equipment

**Figure 4:**
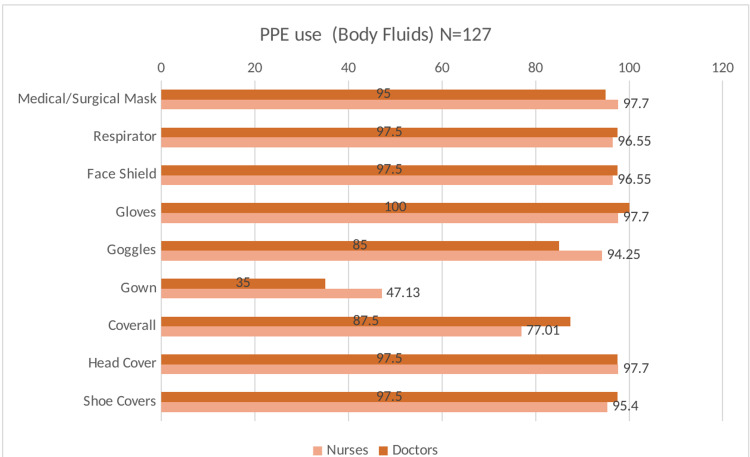
Distribution of utilization of PPE during exposure to body fluid of COVID-19 infected patients PPE: Personal protective equipment

Table 2 presents the data on adherence to infection prevention and control measures in the study participants at baseline. Most participants reported adherence to the recommended hand hygiene measures after touching the patient, before aseptic procedures, after body fluid exposure, and after touching the patient’s surroundings. Furthermore, most participants (99.7%) reported adequate availability of PPE in sufficient quantity at all times in their healthcare facility. The utilization of PPE by the HCWs on COVID-19 duty indicated near-universal use of respirators and face shields with suboptimal use of goggles during face-to-face contact, aerosolizing procedures, and when coming in contact with body fluids (Figures 2,3,4). However, the self-reported adherence to Infection Prevention Control (IPC) measures did not show a statistically significant association with antibody positivity in the HCWs. (Table 3)

**Table 2:** Adherence to Infection Prevention and Control measures at Baseline (N=625) IPC: Infection Prevention Control; PPE: personal protective equipment

Infection Prevention Control measure	Total	Doctors	Nurses
				Hand Hygiene			
Always	539 (87.2)	168 (83.2)	371 (89.2)
Most of the time	71 (11.5)	32 (15.8)	39 (9.4)
Occasionally	7 (1.1)	2 (1)	5 (1.2)
Rarely	1 (0.2)	0 (0)	1 (0.2)
Missing	7(1.1)	1(0.5)	6 (1.4)
Use Alcohol based hand rub before touching patient			
Always	502 (80.6)	155 (76.4)	347 (82.6)
Most of the time	83 (13.3)	40 (19.7)	43 (10.2)
Occasionally	25 (4.0)	5 (2.5)	20 (4.8)
Rarely	13 (2.1)	3 (1.5)	10 (2.4)
Missing	2(0.32)	0	2(0.5)
Use Alcohol based hand rub before cleaning/aseptic procedures			
Always	509 (81.7)	164 (80.8)	345 (82.1)
Most of the time	78 (12.5)	33 (16.3)	45 (10.7)
Occasionally	25 (4.0)	4 (2.0)	21 (5)
Rarely	11 (1.8)	2 (1.0)	9 (2.1)
Missing	2(0.3)	0	2(0.5)
Use Alcohol based hand rub after (risk of) body fluid exposure			
Always	506 (81.2)	170 (83)	336 (80)
Most of the time	71 (11.4)	26 (12.8)	45 (10.7)
Occasionally	26 (4.2)	5 (2.5)	21 (5)
Rarely	20 (3.2)	2 (1)	18 (4.3)
Missing	2(0.3)	0	2(5)
Use Alcohol based hand rub after touching patient			
Always	505 (81.2)	163 (80.3)	342 (81.6)
Most of the time	76 (12.2)	35 (17.2)	41 (9.8)
Occasionally	24 (3.9)	2 (1.0)	22.5.3)
Rarely	17 (2.7)	3 (1.5)	14 (3.3)
Missing	3(0.5)	0	3(0.7)
Use Alcohol based hand rub after touching patient’s surroundings			
Always	495 (79.7)	159 (78.3)	336 (80.4)
Most of the time	77 (12.4)	34 (16.8)	43 (10.3)
Occasionally	35 (5.6)	8 (3.9)	27 (6.5)
Rarely	14 (2.3)	2 (1)	12 (2.9)
Missing	4(0.6)	0	4(0.9)
Follow IPC standard precautions when in contact with any patient			
Always	506 (81.5)	160 (79.2)	346 (82.6)
Most of the time	76 (12.2)	35 (17.3)	41 (9.8)
Occasionally	17 (2.7)	4 (2.0)	13 (3.1)
Rarely	9 (1.5)	1 (0.5)	8 (1.9)
Don’t know	13 (2.1)	2 (1)	11 (2.1)
Missing	4(0.6)	1(0.4)	1(0.4)
Wear PPE when indicated			
Always	568 (91.2)	181 (89.2)	387 (92.1)
Most of the time	31 (5)	18 (8.9)	13 (3.1)
Occasionally	9 (1.4)	1 (0.5)	8 (1.9)
Rarely	15 (2.4)	3 (1.5)	12 (2.9)
Missing	2(0.3)	0	2(0.4)

**Table 3:** Distribution of seroprevalence stratified with adherence to IPC measures at baseline (N=625) IPC: Infection Prevention Control

IPC	SARS-CoV-2 Seropositive	SARS-CoV-2 Seronegative	Unadjusted odds	p-value
	(n=321)	(n=304)		
Hand hygiene				
Always	271(84.4)	268(88.1)		
Most of the time	43(13.4)	28(9.2)	1	
Sometimes	4(1.2)	3(0.9)	1.5(0.9,2.5)	0.25
Rarely	0	1(0.3)	1.3(0.2,5.9)	
Missing	3(0.9)	4(1.3)	-	
Hand hygiene before touching the patient				
Always	255(79.4)	247(81.2)	1	0.15
Most of the time	48(14.9)	35(11.5)	1.3(0.8,2.1)	
Sometimes	14(4.3)	11(3.6)	1.2(0.5,2.7)	
Rarely	3(0.9)	10(3.2)	0.2(0.1,1)	
Missing	1(0.3)	1(0.3)	0	
Hand hygiene before an aseptic procedure				
Always	255(79.4)	254(83.5)	1	
Most of time	47(14.6)	31(10.2)	1.5(0.9,2.4)	0.11
Sometimes	15(4.6)	10(3.2)	1.4(0.6,3.3)	
Rarely	3(0.9)	8(2.6)	0.3(0.1,1.4)	
Missing	1(0.3)	1(0.3)		
Hand hygiene after body fluid exposure				
Always	252(78.5)	254(83.5)	1	
Most of time	45(14)	26(8.5)	1.7(1,2.9)	
Sometimes	15(4.6)	11(3.6)	1.3(0.6,3)	0.11
Rarely	8(2.4)	12(3.9)	0.6(0.2,1.6)	
Missing	1(0.3)	1(0.3)		
Hand hygiene after touching patient				
Always	258(80.3)	247(81.2)	1	
Most of time	45(14)	31(10.2)	1.3(0.8,2.2)	
Sometimes	11(3.4)	13(4.2)	0.8(0.3,1.8)	0.15
Rarely	5(1.5)	12(3.90	0.3(0.1,1.1)	
Missing	2(0.6)	1(0.3)		
Hand hygiene after touching patient’s surrounding				
Always	251(78.1)	244(80.2)	1	
Most of time	45(14)	32(10.5)	1.3(0.8,2.2)	0.54
Sometimes	17(5.3)	18(5.9)	0.9(0.4,1.8)	
Rarely	6(1.8)	8(2.6)	0.7(0.2,2.1)	
Missing	2(0.6)	2(0.6)		
Follow IPC standard precautions				
Always	252(78.5)	254(83.55)	1	
Most of time	44(13.7)	32(10.5)	1.3(0.8,2.2)	
Sometimes	12(3.7)	5(1.6)	2.4(0.8,6.9)	0.35
Rarely	4(1.2)	5(1.6)	0.8(0.2,3)	
I don’t know	7(2.1)	6(1.9)	1.1(0.3,3.5)	
Missing	2(0.6)	2(0.6)		

Seroconversion with SARS-CoV-2 and risk factors

The incidence of seroconversion in the baseline seronegative cohort on follow-up after 21-28 days (median 24 days) was observed in 35 (14.9%) HCWs (n=245). The overall incidence rate of SARS-CoV-2 seropositivity was 5.9 (95% CI 4.2, 8.2) per 1000 person-days. Among the baseline seronegative HCWs on follow-up, complete vaccination with two doses of either Covishield (AZD1222) or Covaxin (BBV152) vaccine was the only factor that was significantly associated with seroconversion indicating the presence of detectable antibodies (p<0.001) (Table 4). Furthermore, the difference in the proportion of HCWs adherence to the IPC measures did not significantly vary among the seropositive and the seronegative subgroups on follow-up. (Table 5)

**Table 4:** Factors associated with seropositivity at follow up (N= 245)* *59 lost to follow-up ILI: influenza-like illness

Characteristic	Total	SARS-CoV-2 Seropositive (Incidence of infection)	Relative Risk (95% C.I)	p-value
	No. (%)			
Age				
Mean (SD)	37.4 (9.8)	-		
Sex				
Male	90 (37.2)	12 (13.3)	0.88 (0.46, 1.68)	0.701
Female	152 (62.8)	23 (15.1)	1	
Occupation				
Doctor	65 (26.9)	11 (16.9)	1.21 (0.56, 2.65)	0.63
Nurse	177 (73.1)	24 (13.6)	1	
Duty duration				
<2 months	178 (73.9)	27 (15.2)	1.07 (0.51, 2.24)	0.849
≥2 months	63 (26.1)	8 (12.7)	1	
History of Covid-19 infection				
Present	10 (4.1)	3 (30.0)	1	0.16
Absent	232 (95.9)	32 (91.4)	2.68 (0.66, 10.69)	
ILI Symptoms				
Present	25 (10.3)	1 (4.00)	1	0.15
Absent	217 (89.7)	34 (15.7	0.26 (0.04, 1.78)	
Smoker				
Yes	5 (2.1)	0 (0)	-	-
No	237 (97.9)	35 (14.8)		
Vaccination status				
Complete	47 (19.2)	17 (36.2)	6.0 (2.7, 13.5)	<0.001
Partial	35 (14.3)	4 (11.4)	1.4 (0.42, 4.45)	
Unvaccinated	163 (66.5)	14 (8.5)	1	

**Table 5:** Distribution of adherence to IPC measures with seroconversion (N=245) *Optimal infection prevention and control measures were defined as adherence to the practice ‘Always’ or ‘Most of the Time’ while non-adherence to IPC was defined as ‘Sometimes’ or ‘Rarely’ IPC: Infection Prevention Control

IPC*	Seropositive	Seronegative	Unadjusted Odds	p value
Hand Hygiene				
Optimal	34 (100)	206 (99.0)	1	-
Sub-optimal	0	2 (1)	1	
Use Alcohol based hand rub before touching patient				
Optimal	31 (91.2)	196 (93.3)	0.74 (0.20, 2.72)	0.648
Sub-optimal	3 (8.8)	14 (6.7)	1	
Use Alcohol based hand rub before cleaning/aseptic procedures				
Optimal	32 (94.1)	198 (94.3)	0.97 (0.21, 4.54)	0.969
Sub-optimal	2 (5.8)	12 (5.7)	1	
Use Alcohol based hand rub after (risk of) body fluid exposure				
Optimal	32 (94.1)	195 (92.9)	1.23 (0.27, 5.64)	0.789
Sub-optimal	2 (5.9)	15 (7.1)	1	
Use Alcohol based hand rub after touching patient				
Optimal	32 (94.1)	193 (91.9)	1.41 (0.31, 6.39)	0.657
Sub-optimal	2 (5.9)	17 (8.1)	1	
Use Alcohol based hand rub after touching patient’s surroundings				
Optimal	32 (94.1)	191 (91.0)	1.59 (0.35, 7.16)	0.545
Sub-optimal	2 (5.9)	19 (9.1)	1	
Adhere to IPC standard precautions when in patient contact				
Optimal	33 (97.1)	197 (94.3)	2.01 (0.25, 15.98)	0.509
Sub-optimal	1 (2.9)	12 (5.7)	1	
Wear PPE when indicated				
Optimal	33 (97.1)	201 (95.7)	1.48 (0.18, 12.05)	0.715
Sub-optimal	1 (2.9)	9 (4.3)	1	

Symptoms suggestive of COVID-19 were non-specific and did not show any statistically significant variation between the SARS-CoV-2 seropositive and the seronegative HCWs at baseline. However, the onset of symptoms was observed in 12 (34.3%, n=35) of the HCWs with evidence of SARS-CoV-2 seroconversion during follow-up assessment. (Table 6)

**Table 6:** Distribution of symptom profile in study participants at baseline and follow-up

	Seronegative (304)	Seropositive (321)
At baseline (N=625) Symptomatic	51 (17.1)	56 (17.6)
Fever	12 (4.0)	13 (4.1)
Sore throat	28 (9.2)	22 (6.9)
Cough	28 (9.2)	30 (9.4)
Runny nose	15 (4.9)	17 (5.3)
Shortness of breath	6 (2)	7 (2.2)
Chills	3 (1)	8 (2.5)
Vomiting	4 (1.3)	0
Nausea	5 (1.6)	3 (0.9)
Diarrhoea	2 (0.7)	5 (1.6)
Headache	23 (7.6)	15 (4.7)
Rash	1 (0.3)	3 (0.9)
Conjunctivitis	2 (0.7)	3 (0.9)
Muscle aches	20 (6.6)	24 (7.5)
Joint ache	16 (5.3)	20 (6.2)
Loss of appetite	7 (2.3)	7 (2.2)
Loss of smell or taste	5 (1.6)	14 (4.4)
Nose bleed	2 (0.7)	4 (1.3)
Fatigue	22 (7.2)	23 (7.2)
Seizures	0	0
	Seronegative (210)	Seropositive (35)
At Follow up (N=245) Symptomatic	24 (11.4)	12 (34.3)
Fever	4 (1.9)	1 (2.9)
Sore throat	12 (5.7)	4 (11.4)
Cough	17 (8.1)	7 (20)
Runny nose	16 (7.6)	4 (11.4)
Shortness of breath	2 (0.95)	0
Chills	2 (0.95)	1 (2.9)
Vomiting	3 (1.4)	0
Nausea	1 (0.5)	0
Diarrhoea	5 (2.4)	3 (8.6)
Rash	1 (0.5)	0
Conjunctivitis	0	0
Muscle aches	1 (0.5)	0
Joint ache	5 (2.4)	0
Loss of appetite	1 (0.5)	0
Loss of smell (anosmia) or taste	1 (0.5)	0
Nose bleed	0	0
Fatigue	4 (1.9)	0
Seizures	1 (0.5)	0

## Discussion

The present study was conducted among healthcare workers (HCWs) comprising doctors and nurses posted for COVID-19 duty at a dedicated tertiary care COVID-19 hospital and medical college complex in New Delhi, India. Nearly one in two (51.4%) participants were seropositive at baseline with antibody positivity unrelated to their sociodemographic or clinical characteristics. Despite high usage of PPE which has proven efficacy in the prevention of SARS-CoV-2 infection, the seroconversion in the seronegative participants at baseline was not associated with the self-reported adherence to the standard infection prevention and control measures.

This is one of the first COVID-19 related cohort studies among HCWs in India that was conducted in the real-world setting of a high burden tertiary care COVID-19 hospital, for detection of antibodies suggestive of past infection to SARS-CoV-2, with a majority of the participants being unvaccinated at baseline. Unlike some population-based seroepidemiological studies, the sex of the participants was not associated with seropositivity, probably since the participants had similar age and occupational profile indicating a behavioural rather than biological predisposition to infection [21-24]. Furthermore, the seroprevalence in the HCWs was lower compared to the estimates observed in the general population of the state of Delhi [24]. However, a nationwide Indian serosurvey in the pre-vaccination period observed the SARS-CoV-2 seroprevalence in doctors to be higher than in the general populations [21]. These findings suggest a complex disease transmission mechanism wherein HCWs despite having sustained exposure to SARS-CoV-2 had comparatively lower rates of infection probably due to high adherence to IPC measures especially involving the use of PPE. Another study in the USA by Jacob et al (2021) also reported the absence of workplace factors as a risk factor for seropositivity while community contact in the HCWs was associated with higher seropositivity [25]. In contrast to a study in Europe, in this study HCWs working in the intensive care unit of the hospital had higher seroprevalence compared to the other departments [11].

There are two key implications of this study. First, HCWs in a high-risk environment nearly universally prefer the utilization of PPE for protection against COVID-19. However, the lack of a statistically significant difference in the incidence of infection in the optimal compared to the sub-optimally adherent HCWs suggest either improper use practice, recall bias, or overestimation of the IPC adherence estimates because of the bias induced from social desirability. A recommended alternative methodology would entail frequent observation by trained observers during COVID-19 patient management to assess adherence to IPC during all the potential moments of exposure and the correct usage of PPE. Second, the evaluation of antibody response in healthcare workers post-vaccination, detection of neutralizing antibodies in seropositive participants, and the possibility of considering booster doses in the absence of adequate immune response warrant urgent consideration in this high-risk cohort.

There are certain study limitations. First, the data collection was mostly completed over a period of four months (December-March) when the COVID-19 disease burden was lower in Delhi compared to the subsequent (April-May) period when disease burden was very high as it coincided with a nationwide second wave period of the pandemic fuelled by mutant strains. Moreover, antibody response to SARS-CoV-2 infection is usually induced 1-3 weeks post-infection so the early screening for antibodies may have generated false negative reports in those participants who were infected but tested prior to their seroconversion. Consequently, the seroprevalence estimates obtained in this study are a likely underestimation. Second, the vaccination in HCWs was an effect modifier as it increased the seroprevalence estimates both at baseline and during follow-up. The WantaiTM kit used was unable to differentiate between natural-infection-induced versus vaccine-induced seroconversion. Finally, the possibility of contraction of infection during off-duty hours during community interaction with other HCWs or family members was not ascertained in this study since most HCWs remained in isolation in dedicated hotels and hostels during post-duty hours and subsequently a week thereafter. However, the possibility of infection from sources other than the hospitalized COVID-19 patients cannot be completely ruled out. 

## Conclusions

Healthcare workers are likely to utilize personal protective equipment (PPE) and follow the IPC precautions when available at their health facilities during the management of COVID-19 patients. High rates of adherence to the use of PPE are also likely to translate into overall lower rates of SARS-CoV-2 infection compared to the general population despite the greater frequency and sustained risk of infection contraction. Healthcare providers providing outpatient services with sustained close contact with patients such as dentists may have a high risk of infection warranting the use of effective PPE in these comparatively low-risk settings. Training of HCWs in the use of PPE may be suboptimal considering the low cumulative training period accorded to the same. Consequently, hospital administrators should provide an enhanced focus on training of HCW for the appropriate use of PPE. 

The evaluation of antibody response in healthcare workers post-vaccination and the possibility of considering booster doses in the absence of immune response warrant consideration in this high-risk cohort. Future SARS-CoV-2 serosurveys in HCWs should identify the effectiveness of vaccines in preventing infection, disease, and severe disease in this high-risk group.
